# How to Implement Clinical 7T MRI—Practical Considerations and Experience with Ultra-High-Field MRI

**DOI:** 10.3390/bioengineering11121228

**Published:** 2024-12-05

**Authors:** Justin Cramer, Ichiro Ikuta, Yuxiang Zhou

**Affiliations:** Department of Radiology, Mayo Clinic Arizona, 5711 E Mayo Blvd, Phoenix, AZ 85054, USA

**Keywords:** MRI, 7 Tesla, 7T, ultra-high field

## Abstract

The implementation of clinical 7T MRI presents both opportunities and challenges for advanced medical imaging. This tutorial provides practical considerations and experiences with 7T MRI in clinical settings. We first explore the history and evolution of MRI technology, highlighting the benefits of increased signal-to-noise ratio (SNR), contrast-to-noise ratio (CNR), and susceptibility at 7T. Technical challenges such as increased susceptibility artifacts and RF inhomogeneity are also discussed, along with innovative adaptations. This review also discusses hardware and software considerations, including new parallel transmission head coils and advanced image processing techniques to optimize image quality. Safety considerations, such as managing tissue heating and susceptibility to artifacts, are also discussed. Additionally, clinical applications of 7T MRI are examined, focusing on neurological conditions such as epilepsy, multiple sclerosis, and vascular imaging. Emerging trends in the use of 7T MRI for spectroscopy, perfusion imaging, and multinuclear imaging are explored, with insights into the future of ultra-high-field MRI in clinical practice. This review aims to provide clinicians, technologists, and researchers with a roadmap for successfully implementing 7T MRI in both research and clinical environments.

## 1. Introduction

Magnetic resonance imaging (MRI) is a sophisticated and non-invasive medical imaging technique that utilizes magnetic fields, radio waves, and computer processing to generate detailed images of organs and tissues within the body [1]. With Food and Drug Administration (FDA) approval of 7T MRI for clinical use in 2017, 7T has transitioned from the research realm into more routine clinical use. This tutorial provides an in-depth exploration of the opportunities and challenges associated with 7T MRI, offering a foundation for understanding its physics, technical considerations, and clinical and research applications.

### 1.1. MR Image Formation Background

Simply put, the MRI scanner forms images by detecting and localizing a signal emitted by hydrogen atoms after manipulation in the MR environment. This section details this process to provide a foundational understanding of MRI.

First, hydrogen nuclei are predominantly used for MRI as opposed to other nuclei due to their abundance in biologic tissues. (Imaging of other nuclei is increasingly feasible at 7T, as discussed later.) Each hydrogen nucleus, or proton, has a positive charge, and is said to possess a “spin”. While not in reality a physical spin like a top, this analogy is useful for understanding MR physics. Recalling Faraday’s law of electromagnetic induction, that spinning, positively charged hydrogen nucleus is essentially a moving charge or current, which induces a magnetic field or tiny magnetic dipole. Normally, hydrogen dipoles are oriented randomly. However, when placed in a strong external magnetic field (referred to as B_0_), the protons align with or against the magnetic field. A slightly greater proportion align with the magnetic field, creating a net signal detected in MR image formation. With increasing magnetic field strength, a greater proportion align with the field, creating more signal which increases in a roughly linear fashion. This phenomenon is the fundamental benefit of higher magnetic field strength [2].

The nuclei are then excited to produce a detectable signal. The nuclei do not align statically with the magnetic field; rather, they move along a path around the magnetic field direction at a certain frequency. This movement is called “precession”, and the frequency of precession is called the Larmor frequency. The Larmor frequency depends directly on magnetic field strength and is discussed further in Section 2.1.1. To excite the nuclei, a radiofrequency (RF) pulse (referred to as B_1_) is transmitted from a coil. This coil may be integrated into the MRI machine or a dedicated device placed over the patient. The RF pulse, tuned to the Larmor frequency, is applied perpendicular to the B_0_ field to tip the net magnetization vector away from the B_0_ axis, exciting the protons. As the excited protons relax back to their equilibrium state, they emit an oscillating electromagnetic signal, which then induces a current in a receiver coil. Like the RF transmitter coil, this receiver coil can either be within the scanner or a separate device. This process, known as nuclear magnetic resonance (NMR), is the foundational principle of MRI [2].

Localization of the received signal created by the magnetic field (B_0_) and RF pulse (B_1_) is the final step in image formation. This relies on hardware in the MRI scanner known as gradient magnetic fields, composed of three orthogonally aligned magnetic fields. A slice selection gradient excites protons in a specific slice (cross-sectional location along the patient’s *z*-axis). Additional frequency and phase-encoding gradients further localize the signal within the selected slice along the x and y axes. The combination of these data is detected by the receiver coil and recorded as “k-space data”. This k-space data can then be encoded into images using a Fourier transform [2]. Variations in various parameters of acquisition result in different sequences, such as T1, T2, diffusion-weighted imaging, and susceptibility-weighted imaging. Notably, MRI does not involve ionizing radiation, a significant benefit.

### 1.2. Other Applications of MRI

Besides structural image formation described above, advancements in MRI have introduced other specialized techniques. Examples include blood oxygen level-dependent (BOLD) functional MRI (fMRI) [3] for mapping brain activity by detecting changes in blood flow and oxygenation levels, magnetic resonance angiography (MRA) [4] for visualizing blood vessels by exploiting the flow of blood, magnetic resonance spectroscopy (MRS) [5] for analyzing tissue chemical composition, and diffusion tensor imaging (DTI) [6] for assessing the microstructural integrity of tissues.

With its capability to provide superior anatomical detail and functional insights without the use of ionizing radiation, MRI has become an indispensable imaging tool in modern medicine. It plays a critical role in diagnosing and planning treatments for various medical conditions, enhancing patient care and management across diverse clinical specialties.

## 2. 7T Technical Considerations

7T MRI, commonly referred to as “ultra-high-field” (UHF) MRI [7,8,9,10], offers several advantages over more traditional high-field (1.5T and 3T) imaging. As alluded to in the Introduction, a primary benefit of moving from 3T to 7T is a roughly linear increase in signal-to-noise ratio (SNR), which allows for some combination of higher spatial resolution, decreased image noise, and faster imaging. Another benefit is that image contrast is also improved at 7T due to increased T1 relaxation time (discussed later). This increased contrast-to-noise ratio (CNR) results in greater delineation of different structures. Finally, magnetic susceptibility, which is the tendency of certain materials to alter the local magnetic field, is increased at 7T. This is both a benefit and a limitation, allowing for more detailed imaging of some atomic elements, but also worsening some image artifacts which must be mitigated. These are the primary driving factors behind 7T adoption [8,10,11,12], and are explored in more detail below.

### 2.1. 7T Physics Considerations

The increased magnetic field strength is accompanied by several changes in MR parameters, including tissue Larmor frequency, RF wavelength, tissue SNR, tissue CNR, tissue susceptibility, chemical shift, and RF effects. These changes bring about both advantageous and disadvantageous outcomes. The following section explores each factor in more detail.

#### 2.1.1. 7T RF Wavelength Considerations

The RF pulses are responsible for exciting hydrogen protons. There are a few factors to consider regarding RF pulses. First, a more homogeneous RF field throughout the imaged tissue will result in better-quality images. Second, RF is the major source of heating from MRI.

With those factors in mind, consider the following changes with 7T. First, the Larmor equation describes the relationship between the magnetic field strength (B_0_) and the resonance frequency (ω) of protons:

ω = γ⋅B_0_

where γ is a constant: the gyromagnetic ratio for protons. At 7T, the Larmor frequency is approximately 300 MHz for protons, compared to 127.74 MHz at 3T and 63.86 MHz at 1.5T. The Larmor frequency can be used to derive the required RF wavelength for 7T based on the following equation:

λ = c/f

where λ is the wavelength (meters), c is the speed of light (approximately 3 × 10^8^ m per second), and f is the RF frequency (hertz). Given the higher frequency at 7T, the wavelength (λ) of the RF waves will be shorter compared to those used in lower-field scanners. For example, a typical Larmor frequency for a 1.5T scanner is around 64 MHz, resulting in an effective wavelength in tissue of 52 cm [13]. At 7T, the effective wavelength is considerably shorter, approximately 12 cm [14].

Several consequences arise from this shorter RF wavelength. First, the RF field is more heterogeneous at 7T. This results in more heterogeneous images in general, which is undesirable. Moreover, because the wavelength is similar to the size of a human head, constructive and destructive interference can occur and cause additional artifacts [14]. Finally, increased tissue heating is a concern at 7T, which is addressed in the Safety Section.

Parallel transmission (pTx) is a technique that addresses these challenges by using multiple independent RF channels to transmit RF pulses [15,16,17]. By carefully controlling the amplitude, phase, and frequency of each channel, pTx enables a more homogeneous B_1_ field, mitigating wavelength effects and ensuring more uniform excitation.

#### 2.1.2. 7T Relaxation Times and Tissue Contrast

T1 and T2 times are fundamental considerations for MRI sequence design and tissue weighting.

T1 is the longitudinal relaxation time [18,19], and it is the time it takes for roughly 63% of the z component of the nuclear spin to recover. Different tissues have different T1 times, which is what allows for image contrast.

T2 is the transverse relaxation time [20,21], and it is the time it takes for the transverse magnetization (x and y component of the nuclear spin) to drop to 37% of the excited peak after the RF pulse is turned off. Similar to T1, tissues have characteristic T2 relaxation times. For instance, fluids typically exhibit longer T2 relaxation times compared to most solid tissues, and are more hyperintense (brighter) due to their slower signal decay.

T1 and T2 relaxation times change with increasing magnetic field strength, altering tissue contrast compared to lower field strengths [22,23,24]. T1 increases as field strength increases [25]. For most biological tissues, empirical measurements suggest that T1 increases approximately as B_0_^1/3^. This increased T1 is a primary factor contributing to improved image contrast (CNR) at 7T.

The impact of magnetic field strength on T2 relaxation is complex and depends on various factors such as tissue iron content [19,26]. When considering the average across all tissue types, T2 relaxation remains relatively stable within the range of field strengths commonly used in clinical MR imaging (0.2T to 3T). However, at 7T, T2 values may exhibit a shortening trend. Example T1 and T2 values for different tissues and magnetic field strengths are provided in Table 1 [22,24,27,28,29,30].

#### 2.1.3. 7T Susceptibility

Increased susceptibility at 7T is a major factor and is both a benefit and limitation. Susceptibility (χ) is a measure of the extent to which a substance becomes magnetized by and locally alters an external magnetic field. Materials that locally weaken the main magnetic field are called diamagnetic, which includes most body tissues. Materials that locally increase the field are called paramagnetic, superparamagnetic, or ferromagnetic, depending on the magnitude of the effect. Common paramagnetic substances include gadolinium (MRI contrast agents) and deoxyhemoglobin (a type of hemoglobin in veins). The susceptibility effect of different tissues and substances is variable.

In general, susceptibility effects and susceptibility differences are increased at 7T. This can have beneficial effects, such as improved tissue contrast. Visualization of strongly paramagnetic substances such as deoxyhemoglobin is also dramatically improved [31]. This offers important benefits for sequences that target paramagnetic substances, such as susceptibility-weighted imaging (SWI), BOLD fMRI, and dynamic susceptibility contrast (DSC) perfusion.

However, when susceptibility differences are too great, it leads to image distortions or excessive signal dropouts (dark spots). This is particularly problematic at air–brain or bone–brain interfaces such as the skull base and paranasal sinuses, and is magnified at 7T relative to 3T. The susceptibility artifact is a major limitation of 7T MRI [31], although strategies exist to minimize susceptibility artifacts:

i Pulse sequence optimization: There are numerous parameters and techniques that can reduce susceptibility for any given sequence. As examples, these include spin-echo imaging, shortening the echo time, decreasing the voxel size, increasing receiver bandwidth, aligning the phase-encoding direction with susceptibility gradients, radial sampling, and parallel imaging [32]. A detailed discussion of these techniques is beyond the scope of this review.
ii Image post-processing techniques: Artificial intelligence (AI) and more conventional image processing algorithms can be applied to reduce artifacts after the scan is acquired [33].
iii Careful patient screening: Patients with certain metallic implants in certain locations may not be suitable candidates for 7T MRI due to excessive susceptibility artifact even if the implant is technically safe.

#### 2.1.4. 7T B_0_/B_1_ Inhomogeneity Mitigation

At 7T, the main magnetic field (B_0_) is more heterogeneous predominantly due to susceptibility, and the RF field (B_1_) is more homogeneous due to the shorter RF wavelength. Different techniques are available to mitigate these limitations.

In addition to the techniques described above, B_0_ homogeneity can be improved by a process called shimming. Shimming is a complex topic implemented multiple ways, to include the installation of hardware around the main magnetic field (passive shimming) and the integration of hardware with the gradient magnetic coils to tailor the magnetic field to individual patients (active shimming) [34,35,36].

B_1_ homogeneity at 7T is most improved by the use of parallel transmission (pTx) technology, which is implemented in the head coil [16,17,37]. The head coil delivers multiple transmit RF pulses (at our institution, from eight sources around the head), which allows the RF field to be shaped into a more homogeneous distribution compared to a single RF pulse source. In addition, pTx reduces tissue heating, as discussed in the Safety Section. This is a relatively new technique to be commercially available, and is a major upgrade in the latest-generation 7T MRIs.

### 2.2. 7T Hardware Considerations

There are multiple physical considerations to 7T MRI. First, the actual MRI machine is quite large. For example, the Siemens Terra.X is 297 cm long, requiring a minimum room size of 65 m^2^ [38]. This is in contrast with a Siemens Magnetom Skyra 3T MRI, which is 173 cm long and requires a minimum room size of 31 m^2^. The equipment powering the 7T MRI also requires more space in an adjacent room. The stronger magnetic field also requires more shielding or a larger room size to limit the magnetic field strength outside the MRI room (zone 4). At our institution, 7T installation coincided with new construction. This was greatly beneficial, as everything from hallway width and height (to allow for scanner delivery) to sitting of the 7T near other clinical scanners could be coordinated.

The 7T bore is also longer and relatively narrow (60 cm compared with more conventional 70 cm). This can increase the likelihood of claustrophobia and limit the maximum patient size.

### 2.3. 7T Software

Optimized software for 7T MRI is designed to handle the specific challenges and capabilities of this ultra-high-field imaging modality, such as advanced shimming techniques discussed above. At our institution, we utilize the vendor-provided control software. It incorporates advanced reconstruction algorithms like deep learning homogeneity correction [39], and compressed sensing to accelerate image acquisition and reduce RF exposure [40]. Deep learning homogeneity correction was a major component in the latest iterations of Siemens 7T MRI scanners and helps with reduction in susceptibility and other heterogeneities.

### 2.4. 7T Safety

7T MRI poses several safety challenges. In general, the challenges are not unique, just magnified compared to 3T. It is essential to be aware of these risks and take appropriate precautions to ensure the safety of patients, staff, and visitors. The following is a review of the major safety considerations that are heightened at 7T. Other considerations that are similar to 3T, such as gradient magnetic field current induction, are not discussed.

i Magnetic field-increased magnetic forces: Any ferromagnetic objects, such as metal implants, jewelry, or surgical clips, will be subject to a magnetic force when they are placed in a strong magnetic field. The attractive magnetic force can be divided into two kinetic forces: the translational force and the torque force. The former is responsible for the displacement of an object within a magnetic field (translation and diversion), and the latter is responsible for the object’s rotational movement [36,41]. The rotational force exerted on a ferromagnetic object in a magnetic field is proportional to the square of the magnetic field strength B_0_, whereas translational forces are proportional to the product of B_0_ and the spatial field gradient (SFG). The SFG is the change in the static magnetic field with distance from the MRI system, which is greater at 7T than at lower field strengths. Thus, a 7T magnetic field exerts at least 2.3 times the translational force and at least 5.4 times the rotational force on an object compared to a 3T field, and likely much more. Therefore, it is even more critical to ensure that patients and staff are free of any ferromagnetic objects, implants, and devices before entering a 7T MRI room. These metallic objects can be pulled towards the magnet or rotate with great force, potentially causing injury.
ii Magnetic field interaction with electronics: The strong magnetic field can interact with certain implanted devices, leading to malfunction or interference. This is especially concerning for patients with pacemakers, defibrillators, or other electronic implants. While some devices are clear for use at 1.5T or 3T, manufacturer instructions should always be reviewed prior to use in a 7T field.
iii Magnetic field-increased Lenz forces: When a conducting material, even if it is not ferromagnetic, moves through a changing magnetic field, an electrical current is induced within it. This induced current, in turn, generates additional magnetic fields that oppose the object’s motion [42]. This phenomenon, known as Lenz’s law, results in Lenz forces that resist the movement of the object. These Lenz forces are significantly greater when the object moves through the stronger 7-tesla SFG compared to its motion through weaker 1.5- or 3-tesla SFGs. This is a consideration even for safe implants such as orthopedic implants. Very slow table movement in and out of the MRI bore is the primary strategy to mitigate Lenz forces [43]. The 7T table always moves slowly to account for this issue as well as bioeffects discussed below.
iv Magnetic field-increased biologic and physiologic effects: Patients undergoing 7T MRI may experience worsened biologic and physiologic effects due to motion through the high magnetic field. These effects can include vertigo, dizziness, false feelings of motion, nausea, nystagmus, magnetophosphenes, and electrogustatory effects [44,45,46,47,48]. In some cases, patients may also experience discomfort or pain, particularly in areas with high concentrations of nerves or blood vessels. The primary way to mitigate these effects is to limit patient motion during scanning. First, the scanner table moves very slowly. Second, patients are coached to limit head motion until they are off the table, as head rotation can also induce physiologic effects. Anecdotally, this has not been a significant limiting factor for 7T imaging at our institution.
v RF transmission heating: The major safety concern with the RF pulse is tissue and implant heating. Specific absorption rate (SAR) is the metric MRI scanners monitor to determine how much energy has been deposited in tissues during the scan, and it is closely monitored for all magnetic field strengths. However, RF heating is significantly greater at 7T. This is due to several factors, including the shorter RF wavelength being more absorbed in tissue and associated reduced tissue penetration, as well as a greater overall RF energy requirement to due higher flip angles required with increasing magnetic field strength. Due to the shorter RF wavelength in tissues at 7T, smaller metallic objects, implants, and foreign bodies are also more susceptible to heating and potential thermal injury during 7T MRI [49,50]. The primary method of mitigating this heating effect is distance from the imaging coil. At our institution, we require most implants and non-removable metallic external devices to be located >30 cm from the head coil to qualify for 7T. Finally, the newer parallel transmit (pTx) head coils have the potential to reduce RF heating as well by creating a more optimized RF field [17].
vi Claustrophobia: Our 7T MRI has a 60 cm bore. This is a very common bore size for even 3T MRI, with 70 cm considered “large bore”. However, the 7T bore is also longer. In theory, the longer bore could contribute to an increased incidence of claustrophobia. While not formally tracked at our institution, anecdotally this has also not been a major limitation.

Due to these unique considerations, it is essential for each institution to develop a customized safety protocol for 7T prior to implementation. The increased magnetic field strength and increased RF heating are the primary concerns that are significantly different at 7T.

## 3. 7T Structural Imaging and Clinical Applications

**Figure 1:**
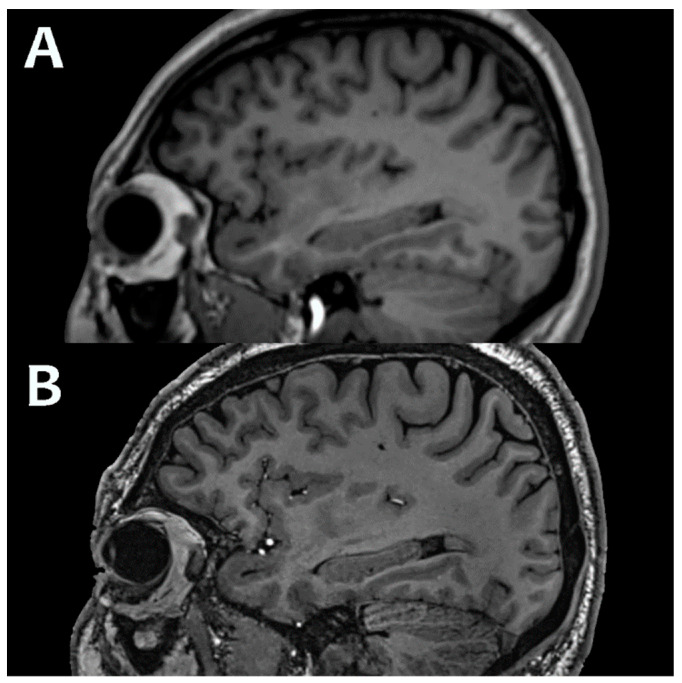
Typical 3D T1 sequence, acquired at 1.0 × 1.0 × 1.0 mm^3^ isovoxel spatial resolution at 3.0T (**A**) and 0.6 × 0.6 × 0.6 mm^3^ at 7T (**B**). Images acquired at the Mayo Clinic.

As discussed, 7T MRI offers several advantages over 3T [51]. The roughly linear increase in signal allows for better (high signal), smaller (higher spatial resolution), and faster imaging (reduced scan time). For example, the 3D T1 sequence can be acquired at 0.6 mm instead of the 1.0 mm typical for 3T (Figure 1), and spatial resolution on other sequences can reach as low as 0.2 mm. The increased CNR allows for better delineation of different tissues, which is crucial for identifying and delineating pathology. Finally, the increased susceptibility effect also increases image contrast, and particularly benefits sequences such as SWI, DSC perfusion, and BOLD fMRI.

The increased susceptibility effect also poses a major challenge, and causes signal loss and distortion near air and bony structures such as the skull base and posterior fossa. Newer technologies such as the pTx head coil help to mitigate this effect, but their use is still a work in progress [17]. 7T is also more prone to motion artifact, largely due to higher spatial resolutions, which complicates imaging of certain structures such as the eyes.

Another limitation is that currently only head and knee coils are approved by the U.S. Food and Drug Administration (FDA) and reimbursed financially, leading to a lack of interest in imaging of the neck, chest, and abdomen. Currently, most clinical 7T imaging is neurologic and intracranial, with epilepsy, multiple sclerosis (MS), and vascular pathologies representing the most common indications.

**Figure 2:**
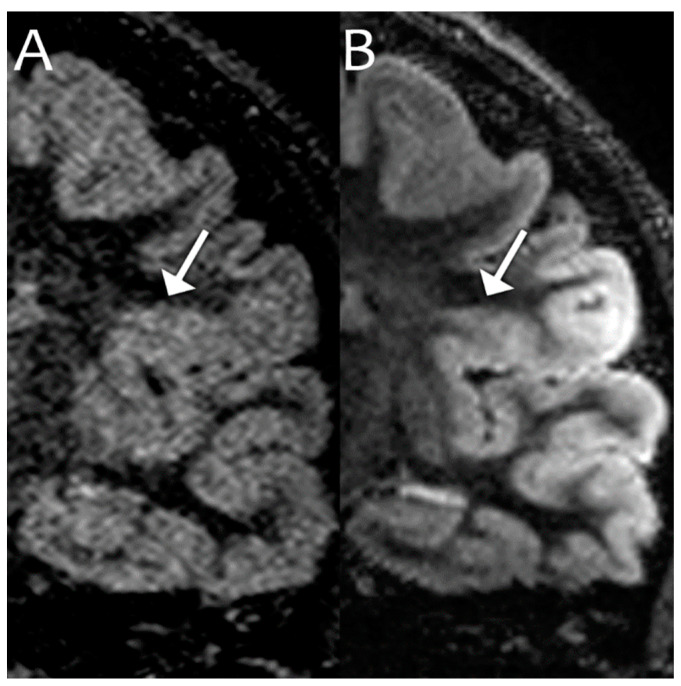
Coronal dual inversion recovery (DIR) images on the same patient performed at 3T (**A**) and 7T (**B**), which can be viewed similar to a T1 sequence. Note how the flame-shaped left frontal FCD (white arrows) is better delineated by the 7T image due to decreased noise, increased spatial resolution, and increased contrast resolution. Images acquired at the Mayo Clinic.

For epilepsy, the major goal is to find underlying malformations of cortical development (MCD) such as focal cortical dysplasia (FCD), as their identification can greatly improve epilepsy surgery outcomes [52]. These are often very subtle foci of blurring at the cerebral cortex–white matter junction (Figure 2). The lower noise, increased spatial resolution, and increased contrast resolution at 7T are particularly beneficial for this indication.

**Figure 3:**
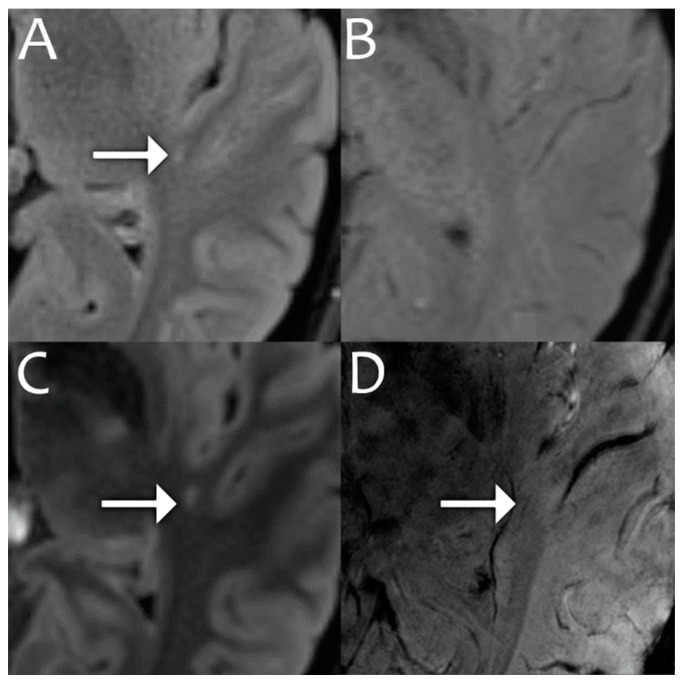
3T FLAIR (**A**) demonstrates a hyperintense lesion (white arrow) with no definite central vein on 3T SWI (**B**). 7T FLAIR (**C**) demonstrates a hyperintense lesion (white arrow) with a corresponding central vein on 7T SWI (**D**), which is more suggestive of an MS demyelinating lesion. Images acquired at the Mayo Clinic.

There are a few advantages to 7T with MS. First, more demyelinating lesions can be detected, particularly cortical lesions [51], which has important prognostic implications. Second, there is improved detection of the central vein sign (CVS), which is a vein running through a white matter lesion (Figure 3). The CVS is highly suggestive of MS and helpful for confirming the diagnosis, with sensitivity and specificity of up to 100% [53]. Finally, paramagnetic rim lesions, which aid with diagnosis and prognosis, are identified more readily at 7T [54].

**Figure 4:**
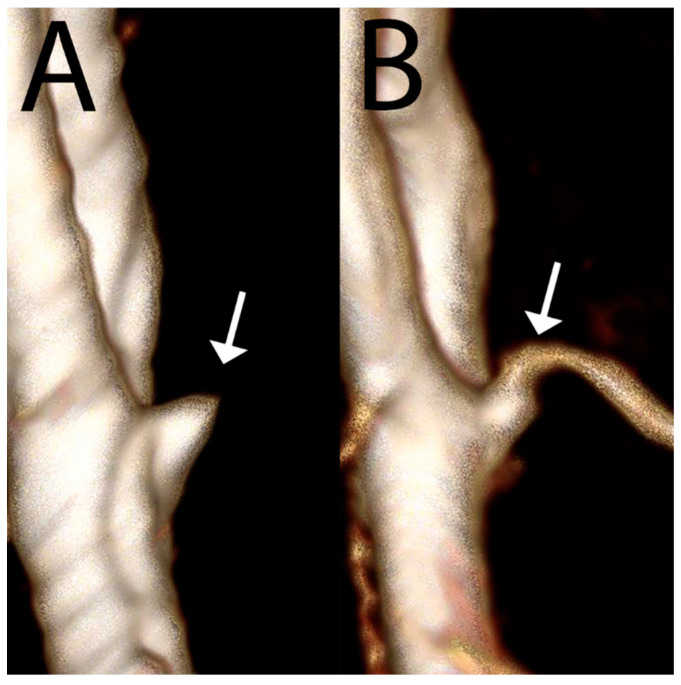
Three-dimensional reformats of an MRA performed at 3T (**A**) raised concern for an aneurysm arising from the anterior communicating artery (white arrow). Repeat MRA at 7T (**B**) clearly demonstrates this structure as a normal infundibular origin to an anterior communicating artery branch vessel, not an aneurysm. Since this is an infundibulum, this patient will not need life-long imaging surveillance or neurosurgery. Images acquired at the Mayo Clinic.

The increased spatial resolution and contrast of 7T also greatly benefit MRA, allowing for the visualization of smaller vessels. This is very helpful in differentiating infundibula (normal vessel origins) from pathologic aneurysms (Figure 4), which require surveillance and/or treatment and are understandably a stressor for patients [55].

While these are the most common indications for 7T MRI, numerous brain pathologies may potentially benefit. Head imaging, including structures such as the temporal bones, pituitary, and orbits pose greater difficulties due to their location within osseous structures and associated susceptibility artifact. With improving 7T hardware technology such as the pTx head coil, imaging of these structures at 7T may also become routine.

## 4. 7T Advanced Imaging Protocols

**Figure 5:**
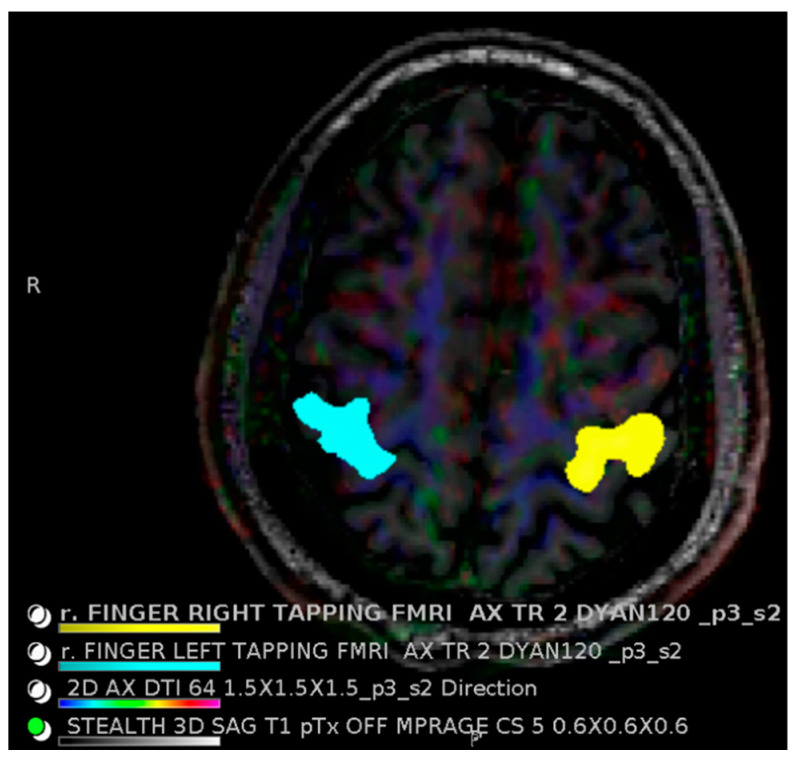
Functional MRI at 7 Tesla provides more than twice the usual BOLD signal and is localized more closely to the center of activation. This healthy volunteer shows finger tapping (left finger in blue and right finger in yellow) localizing to the appropriate anatomic region. Images acquired at the Mayo Clinic.

Pre-surgical fMRI helps localize eloquent brain functions such as language and sensorimotor areas, which are important to preserve for patient function and improved survival [56]. Both resting state and task-based fMRI demonstrate increased signal at 7T relative to 3T with BOLD signal-to-noise ratio roughly proportional to B_0_^2^ [57]. This is vital given the small signal changes under evaluation via BOLD imaging and may lead to abbreviated sequences with faster scan times. Localization may also be more accurate, with task-based centers of activation more closely localized to a particular cerebral gyrus at 7T (Figure 5), and 3T sometimes showing inaccurate task activations across two cerebral gyri and an intervening sulcus (space filled with cerebrospinal fluid that does not generate brain activity) [58].

The same BOLD sequence can be used to evaluate cerebrovascular reserve (CVR) [59], which is a measure of the brain’s capacity to increase blood flow in response to increased metabolic demand. Increasing blood carbon dioxide levels causes a diffuse arterial dilation, usually producing a diffuse increase in brain BOLD signal [60]. A patient’s intra-arterial carbon dioxide levels can be artificially elevated by use of a breath hold [61], administration of a carbonic anhydrase inhibitor drug like acetazolamide [62], or through a breathing mask with careful titration of oxygen and carbon dioxide levels [63]. These CVR evaluations become useful to detect false negative fMRI results (such as near tumors that secrete chemicals causing constant maximal vasodilation, preventing BOLD imaging detection of regional blood flow differences) [64], or to evaluate for critical stenoses that can cause a “steal” phenomenon with blood flow decreasing during stressful situations [65]. This information can alter neurosurgical treatment decisions.

Traditional ^1^H proton MRS also benefits from the ultra-high-field strength of 7T, enabling the resolution of a greater number of metabolite peaks and improved spectral resolution compared to 3T systems [66]. The quantitatively narrower full width at half maximum (FWHM) at 7T allows for more metabolites to be identified with higher confidence [67]. While proton MRS can help diagnose tumors, demyelination, stroke, and toxic/metabolic disorders, “X-nuclei” refers to non-proton-based MR spectroscopy. Some X-nuclei elements were previously difficult to image at 3T due to the very low concentrations in the body (relatively to the highly abundant hydrogen protons). However, 7T significantly improves sensitivity and resolution for X-nuclei imaging. For example, our group has a 23Na sodium coil to evaluate tissue sodium content (TSC) for tumors, demyelinating diseases, and neurodegenerative disorders [68,69]. Another promising X-nuclei imaging is phosphorous (31P) for brain tumor pH measurements as a biomarker for treatment monitoring [70].

MR perfusion has been employed for the evaluation of tumors for quite some time, although it is relatively new at 7T. While spatial resolution of DSC perfusion at 3T is approximately 5 mm, our initial experience with 7T DSC is at a resolution of 1.5 mm isotropic. This improved spatial resolution will allow for superior assessment of post-treatment effects.

Gadolinium-based and iron-based contrast agents are not yet FDA-approved for use with clinical 7T MRI, although research and clinical exams with reimbursement continue [71].

## 5. 7T Future Directions

From a hardware perspective, there are numerous future directions for 7T MR brain imaging. The latest generation of 7T scanners contains several upgrades to address traditional shortcomings of 7T including susceptibility, magnetic field inhomogeneity, and motion. These upgrades include more powerful gradients which speed up imaging and reduce motion artifact, a pTx head coil that reduces susceptibility and RF inhomogeneity [37], and AI-based image processing for both acceleration and artifact reduction. Further refinement along these lines can be expected in the future. For example, researchers at UC Berkeley have developed a next-generation 7T scanner with upgrades to the gradient system and head coils to further increase image quality [72].

Clinically, as more 7T scanners are installed with the intent of clinical utilization, the breadth of 7T use for brain imaging will be increased. With increased spatial and contrast resolution, many new imaging findings will be explored and reported; for example, differing rates of incidental pituitary gland findings at 7T versus 3T [73]. Higher image quality can also lead to improved surgical outcomes; for example, when used for preoperative deep brain stimulator planning [74]. Also with expanding indications, expect the adoption of techniques well-established at 3T that are relatively under-utilized at 7T; for example, perfusion imaging. Finally, the head coil also allows for imaging of head structures such as the sella, orbits, face, and temporal bones. Traditionally, imaging of these structures at 7T has been very limited, but this may expand with the latest generation of scanners that decrease susceptibility.

As discussed in the advanced imaging section, 7T also conveys advantages to spectroscopy, multinuclear imaging, and functional MRI. Functional MRI has well-established clinical indications at 3T, and routine clinical use at 7T is likely. There is much research interest and promise in spectroscopy and multinuclear imaging, and the increased resolution of ultra-high field MR spectroscopy could lead to wider use in clinical evaluations.

Outside of the head, 7T imaging of the knee is already FDA-approved. While the same image quality gains translate to musculoskeletal imaging, 7T has not gained as much traction in this space, and will continue to be an area of refinement and development [75]. There is active development of coils for imaging of nearly every other body part including body, breast, and neck [76,77]. These will be growth areas for future 7T imaging.

## 6. Summary

7T MRI has made significant strides, demonstrating clear clinical benefits and opening new avenues for research. While the ultra-high-field strength presents logistical and technical challenges, advancements in medical physics continue to mitigate these issues, though limitations remain. There is tremendous future opportunity for clinical and research advancements.

## Figures and Tables

**Table 1:** T1 and T2 relaxation times at different field strengths.

	1.5T		3.0T		7.0T	
	T1 (ms)	T2 (ms)	T1 (ms)	T2 (ms)	T1 (ms)	T2 (ms)
White Matter	600–700	80	850–950	75	1250–1350	70
Gray Matter	950–1100	100	1615	95	2065	90
CSF	4500	220	5500	200	>6000	1000
Muscle	900	50	1300	40	1750	40
Fat	250	60	310	55	400	50
Blood	1200	100~200	1900	275	2500	130
